# What is the best way to manage ureteric calculi in the time of COVID‐19? A comparison of extracorporeal shockwave lithotripsy (SWL) and ureteroscopy (URS) in an Australian health‐care setting

**DOI:** 10.1002/bco2.55

**Published:** 2020-11-07

**Authors:** Matthew Farag, Gregory S. Jack, Lih‐Ming Wong, Damien M. Bolton, Daniel Lenaghan

**Affiliations:** ^1^ Department of Urology University of Melbourne Austin Health Heidelberg VIC Australia; ^2^ Department of Urology St Vincent’s Melbourne Melbourne VIC Australia

**Keywords:** extracorporeal shockwave lithotripsy (SWL), laser lithotripsy, renal colic, ureteric stones, urolithiasis

## Abstract

**Objectives:**

To determine the best way to intervene for ureteric stones which still require treatment during the COVID‐19 pandemic, with respect to infection control. In this setting, in which resources are constrained, extracorporeal shockwave lithotripsy (SWL) has prima facie advantages over ureteroscopy (URS). It is also necessary to also consider posttreatment resource consumption in regards to complications and repeat procedures.

**Subjects and methods:**

The ideal ureteric stone treatment during a pandemic such as COVID‐19 would involve minimum resource consumption and a minimum number of patient attendances. We compared all patients initially treated with SWL to those initially treated with URS for acute ureteral colic within the state of Victoria, Australia in 2017.

**Results:**

A total of 2724 ureteric stones were analyzed, a cumulative “3‐month exposure and burden on the healthcare system” was calculated for each patient by their initial procedure type. The readmission rate for URS was significantly higher than for SWL, 0.92 readmissions/patient for URS versus 0.54 readmissions/patient for SWL (*P* < .001). The cumulative hospital stay per patient for these two procedures was 2.35 days for SWL versus 3.21 days for URS (*P* < .001). The number of procedures per patient was 1.52 for SWL versus 1.89 for URS (*P* = .0213).

**Conclusions:**

Patients with ureteric stones treated initially by SWL have shorter length of stay with fewer overall attendances and procedures at 3 months than those treated with URS. During a pandemic such as COVID‐19, SWL may have benefits in preserving hospital resources and limiting opportunity for virus transmission, compared to URS.

## 1 INTRODUCTION

Ureteric calculi that require treatment can be surgically managed by either ureteroscopic lithotripsy (URS) or extracorporeal shockwave lithotripsy (SWL).^1^, ^2^ Surgical treatment (including URS or SWL) should be offered to adults with ureteric stones and renal colic within 48 hours of diagnosis or readmission, if pain is ongoing and not tolerated or the stone is unlikely to pass.^3^ COVID‐19 is a contagious viral infection, primarily infecting the pulmonary system with respiratory symptoms similar to those seen in the previously reported SARS epidemic in 2003.^4^ Respiratory droplets and close contact transmission are the main routes of transmission hence producing significant risk to those present in the operating theater during procedures involving endotracheal intubation and general anesthesia.^4^ SWL is commonly performed with oral analgesia, thereby limiting infectious respiratory disease transmission, such as COVID‐19.^5^ It is less common for URS to be attempted without anesthesia, due to the more invasive nature of the procedure.^6^

Furthermore, recent analysis has detected the presence of the SARS‐CoV‐2 coronavirus in all of the urine samples of infected patients, this study noted that none of the patients exhibited any signs of urinary symptoms; nine patients were in the initial stage of infection based on clinical assessment.^7^ This high risk to medical professionals has prompted cancelations of nonurgent elective surgeries including urological procedures in Australia and around the world; in addition appropriate personnel protection equipment (PPE) is paramount in different zones of the operation room when procedures are urgent.

The European Association of Urology (EAU) has published guidelines that during the COVID‐19 pandemic, urgent ureteric stones should be temporized with a ureteric stent, preferably placed at the bedside.^8^ This approach is sensible in the setting of a critically overburdened health‐care system. In that situation the choice between URS and SWL is not immediately relevant.

However, at different times during the pandemic, many health‐care systems are operating without excessive workloads, with suppressed numbers of community COVID‐19 cases.^9^ In this situation, it seems reasonable to definitively treat the ureteric stone and thereby avoid stent‐related morbidity and later complications (stent encrustation, urinary tract infections, and colic pain/bladder irritation).^10^ At the same time, it remains paramount to conserve PPE supplies and reduce patient attendances, and hence, potential COVID‐19 transmission to and from health‐care workers. Therefore, the question remains for urologists as to how to most efficiently treat ureteric stones over the course of the COVID‐19 pandemic. SWL has advantages over URS, with no operating team or body fluid exposure, potentially less need for anesthesia and airway manipulation, and therefore, in the first instance fewer health‐care workers involved and less PPE required.

This current study seeks to look more broadly at the real post‐procedure care requirements, in order to ascertain if the advantages of SWL are maintained when subsequent emergency department presentations, further procedures and hospital admissions are considered. Follow‐up imaging in these patients occurred via ultrasound, plain x‐ray, or computer tomography scan (ultra‐low dose when appropriate).^11^

Both SWL and URS continue to evolve with the implementation of new technology. There is variation in published prospective studies that compare SWL to URS; some studies measure the stone‐free rates after the initial procedure as a primary outcome, while others after additional subsequent procedures.^12^ One study found that for stones <10 mm in diameter, there was no significant difference in stone‐free rates between URS and SWL, however, URS was more effective for stones >10 mm.^6^ In terms of cost, URS is significantly more expensive than SWL, It is also more common for a stent to be inserted during URS, which would accrue a cost in also removing the stent. A recent systematic review concluded that at a population level, first‐line SWL should be the first choice treatment for ureteric stones <10 mm.^12^

## 2 METHODS

### 2.1 Study design

We analyzed hospital admission data from the Victorian Admitted Episodes Data set (VAED) managed and audited by the Department of Health and Human Services Victoria, Australia. We analyzed every ureteric stone diagnostic code and treatment code performed in private and public hospitals across Victoria, Australia from January 1, 2017 to December 31, 2017. This complete data set contains de‐identified information for every patient admission within a Victorian public or private hospital; each patient ID is preserved and linked to any subsequent interaction with the health‐care system. Included in this data (but not limited to) is patient demographics, International classification of disease (ICD) coding, Medicare Benefit Schedule (MBS) rebate coding for procedures, anesthesia codes, diagnostic codes, mode of admission, length of inpatient stay, and intensive care requirements. Every patient had an Index Stone Treatment (IST) with either SWL or URS in 2017. Follow‐up admissions (planned and unplanned) and emergency department presentations for every patient were obtained for 3 months post their procedure, this was made possible through linkage with the Victorian Emergency Minimum Data set (VEMD).

### 2.2 End points

Stone complications were defined as any stone‐related admission with renal colic, urinary tract infection, or requiring stone‐related surgery within 3 months. A cumulative “3‐month exposure and burden on the healthcare system” was calculated for each patient by their initial procedure type, this incorporated overall length of stay, emergency presentations, number of readmissions, and subsequent procedures required.

### 2.3 Statistical analysis

Calculations were performed using Stata/MP version 13.0 for Mac (StataCorp LP). Variables were checked for skewness and kurtosis to determine normality. Clinical and demographic features are presented as medians [interquartile range] and means (± standard deviation) for nonparametric and parametric data, respectively. Differences between continuous parametric variables were examined with the *t* test; the Wilcoxon rank‐sum test or the Wilcoxon‐Mann‐Whitney test were used for non‐normally distributed continuous and ordinal variables, while differences between dichotomous variables were evaluated with the χ^2^ test or the Fishers exact test (Tables 1, 2, 3). *P*‐values throughout the results were two sided.

**TABLE 1:** Descriptive statistics

	URS	ESWL	*P*‐value
Number in Cohort	2567	157	
Median age	47 [34‐59]	47 [32‐58]	.8441
Gender (Male) %	73%	71%	.3372
LOS (days)	1.52	1.10	.0003
General anesthesia %	97%	89%	<.001
ICU admission %	0.62%	0.64%	.9832
ASA grade (median [IQR])	2 [1‐3]	2 [1‐3]	.8771

**TABLE 2:** Emergency presentations and readmissions 3 months postoperatively

	URS N = 2567	ESWL N = 157	*P*‐value
*Emergency presentations 3 months (N)*	190	11	
Incidence %	7.40%	7.01%	.0923
UTI/Sepsis	2.07%	0.64%	.3691
Renal colic	5.33%	6.37%	.5832
Length of stay in ED (mins)	264.1	299.8	.4463
Admitted from ED	62.45%	64.68%	.3767
*Readmissions 3 months (N)*	2374	86	
Incidence	92.48%	54.77%	<.001
Average readmissions per patient	0.92	0.54	<.001
% Readmissions 28 days	65.17%	39.23%	<.001
Average LOS at readmission	1.69	1.25	.0964
ICU required during readmission %	0.80%	0%	.2599
Patients requiring another procedure	89.01% (2285/2567)	33.12% (52/157)	<.001

**TABLE 3:** Summary of cumulative exposure/resource consumption per patient over 3 months

	URS	ESWL	*P*‐value
Average number of Hospital attendances per patient	1.99	1.62	.0412
Average number of procedures per patient	1.89	1.52	.0213
Average cumulative LOS per patient (days)	3.21	2.35	<.001

## 3 RESULTS

A total of 2724 ureteric stones were treated in Victoria between January 1, 2017 to December 31, 2017, 94% of patients were treated by URS and 6% by SWL (2567 vs 157). Of these, 1419 cases were treated in private hospitals versus 1305 public hospitals. Further breakdown showed that for URS procedures 1373 were conducted in private versus 1194 in public; SWL patients had 46 procedures in private and 111 in public hospitals. For URS patients 97% received general anesthesia versus 89% of SWL patients, (*P* < .001). About 71% of patients undergoing SWL were male versus 73% of patients undergoing URS (*P* = .337). The median age for both SWL and URS patients was 47 years old (*P* = .844). Mean length of stay (LOS) for URS procedures was significantly longer than for SWL procedures, 1.52 days versus 1.10 (*P* = .0003). Furthermore 76% of SWL patients (120/157) were discharged home on the same day as their procedure, compared to 38% of URS patients (975/2567), (*P* < .0001).

In the 3 months following the initial ureteric stone treatment, we observed 201 emergency presentations relating to renal colic, UTI with/without fever, hydronephrosis, and other stone‐related symptoms. Of those, 11 corresponded to SWL patients (incidence 7.01%), and 190 for URS patients (incidence 7.40%), (*P* = .092) (Table 2). The median triage category was three for both groups (*P* = .3167), and the mean time spent in ED was 299.82 minutes for SWL versus 264.09 minutes for URS (*P* = .446). These unplanned presentations mainly related to renal colic or UTI as the primary diagnosis. For SWL, 91% of presentations related to renal colic versus 9% for UTI; and for URS patients, 72% were for renal colic versus 28% for UTI (*P* = .083). At presentation to emergency, 62.45% of SWL patients required admission versus 64.68% URS patients (*P* = .3767). Hence, the incidence of readmissions through the emergency department was 4.74% for URS and 4.34% for SWL, *P* = .055.

For this cohort of patients there was a total of 2460 elective and emergency readmissions in the 3 months following the initial procedure; an incidence of 54.77% (86/157) for SWL and 92.48% (2374/2567) for URS (*P* < .001) (Table 2). For SWL 39% of these were within 28 days compared to 65% for URS (*P* < .001) (Table 2). The readmission rate per patient was significantly higher for URS than for SWL, 0.92 versus 0.54 (*P* < .001).

Furthermore 89.01% (2285/2567) of URS patients required a subsequent procedure versus 33.12% (52/157) of SWL patients (*P* < .001) within 3 months. For SWL these comprised 51.92% (27/52) for flexible cystoscopy and stent removal, 9.62% (5/52) required a stent, 30.77% (16/52) had further URS or SWL for residual calculi, and 7.69% (4/52) requiring another intervention; with 75% same day discharge (39/52). For URS 66.21% (1513/2285) were for flexible cystoscopy and stent removal, 2.23% (51/2285) required a stent, 30.55% (698/2285) had further URS or SWL for residual calculi, 75% same day DC (1781/2285). At readmission the mean LOS was 1.69 days for URS versus 1.25 days for SWL (*P* = .0964).

A cumulative “3‐month exposure and burden on the healthcare system” was calculated for each patient by their initial procedure type (Table 3). The number of hospital attendances per patient was 1.62 days for SWL versus 1.99 days for URS (*P* = .0412). The number of procedures per patient was 1.52 for SWL versus 1.89 for URS (*P* = .0213). Finally, the cumulative hospital stay per patient for these two procedures was 2.35 days for SWL versus 3.21 days for URS (*P* < .001).

## 4 DISCUSSION

For the patient requiring definitive treatment of a ureteral stone, URS and SWL are the two most commonly used treatment modalities. The current AUA stone management guidelines based on a 2012 Cochrane review, recommend that SWL is the procedure with the least morbidity and lower complication rate compared with URS for the treatment of a single ureteric calculus.^13^ Our large population study further supports this over a 3‐month period following surgery; this is relevant in the setting of a pandemic where resources need to be conserved and contact between patients and health‐care workers needs to be minimized.

Health‐care workers performing physical examinations or are exposed to a patient during aerosol generating procedures such as endotracheal intubation are more likely to contract COVID‐19 than those without such exposures.^14^, ^15^ The first‐generation lithotripter used for SWL required general anesthesia for treatment, however, current generations can be used with a variety of anesthesia techniques, ranging from general anesthesia to just oral analgesia.^16^ In our study, the rate of intubation was significantly higher in URS patients (97%) compared to SWL patients (89%), (*P* < .001); this rate can be reduced further for SWL in a pandemic.

Hospitals in the early outbreak of the pandemic began to struggle to obtain appropriate equipment such as N95 respirators and personal protective equipment. Subsequently the surgical capacity of hospitals was reduced and elective surgeries were canceled and postponed.^17^ The PPE that remains must be used judiciously for urgent procedures such as urgent ureteric stone surgery. Hence, the higher number of hospital attendances per patient for URS, 1.99 days versus 1.62 (*P* = .0412), as well as the higher number of procedures per patient for URS, 1.89 versus 1.52 (*P* = .0213), are important factors that favor SWL for ureteric stone treatment in a pandemic. In the United States, The American College of Surgeons suggested that the more surgery centers decreased interactions and increase the capacity of the medical system to handle testing evaluation and treatment, the better off the United States will be.^17^ In our population, the data suggests that SWL involves fewer interactions per patient with the health‐care system as well as reduced cumulative LOS within 3 months of ureteric surgery, 3.21 days for SWL versus 2.35 days for URS (*P* = <.001).

## 5 LIMITATIONS

This study is retrospective and un‐randomized, however, it includes all patients treated for ureteric stones in the population of Victoria, Australia. Stone characteristics, including size and location, were not included in this Department of Health data set and while the demographics of the two cohorts are well matched, it is possible that stone characteristics were significantly different. There are no local guidelines to direct the choice between SWL and URS based on stone size or position. In this population the choice of approach is predominantly determined by the availability of equipment (lithotripter or ureteroscope/laser) rather than clinical factors.

There was a relatively high rate of general anesthesia (89%) with SWL in this study compared to other reported series.^5^ While there is little evidence for this impacting treatment success; consideration should be taken when looking to generalize the findings of this study to other health systems or to a COVID‐19 setting, where GA will be avoided whenever possible.

In practice, some ureteric stone patients will not be suitable for SWL (eg, cases of severe obesity or uncorrected coagulopathy).^18^ However, for most ureteric stones, the decision between URS and SWL comes down to a variety of considerations, and the overall resource consumption and extent of exposure, as considered in this study, may often be decisive during a pandemic. Uric acid stones (up to 10% of renal stones) are radiolucent, and therefore, not easily targeted for SWL, but these are likely to be most efficiently treated with medical dissolution therapy rather than SWL or URS.^19^

## 6 CONCLUSION

This population study suggests that SWL requires less health‐care resources than URS over a 3‐month period for the management of ureteric stones requiring urgent treatment. This information is useful during a pandemic such as the COVID‐19 outbreak where the aim is not only to clear ureteric stones, but also to conserve PPE and reduce the opportunities for infectious disease transmission.

## CONFLICT OF INTEREST

Dan Lenaghan, Matthew Farag, Lih‐Ming Wong: None; Damien Bolton: Boston Scientific, Investigator Grant; Gregory Jack: Boston Scientific, lecture honoraria.
